# Different Contributions of Clathrin- and Caveolae-Mediated Endocytosis of Vascular Endothelial Cadherin to Lipopolysaccharide-Induced Vascular Hyperpermeability

**DOI:** 10.1371/journal.pone.0106328

**Published:** 2014-09-02

**Authors:** Ye Zhang, Lianyang Zhang, Yang Li, Shijin Sun, Hao Tan

**Affiliations:** State Key Laboratory of Trauma, Burns and Combined Injury, Institute of Surgery Research, Daping Hospital, Third Military Medical University, Chongqing, China; Thomas Jefferson University, United States of America

## Abstract

Vascular hyperpermeability induced by lipopolysaccharide (LPS) is a common pathogenic process in cases of severe trauma and sepsis. Vascular endothelial cadherin (VE-cad) is a key regulatory molecule involved in this process, although the detailed mechanism through which this molecule acts remains unclear. We assessed the role of clathrin-mediated and caveolae-mediated endocytosis of VE-cad in LPS-induced vascular hyperpermeability in the human vascular endothelial cell line CRL-2922 and determined that vascular permeability and VE-cad localization at the plasma membrane were negatively correlated after LPS treatment. Additionally, the loss of VE-cad at the plasma membrane was caused by both clathrin-mediated and caveolae-mediated endocytosis. Clathrin-mediated endocytosis was dominant early after LPS treatment, and caveolae-mediated endocytosis was dominant hours after LPS treatment. The caveolae-mediated endocytosis of VE-cad was activated through the LPS-Toll-like receptor 4 (TLR4)-Src signaling pathway. Structural changes in the actin cytoskeleton, specifically from polymerization to depolymerization, were important reasons for the switching of the VE-cad endocytosis pathway from clathrin-mediated to caveolae-mediated. Our findings suggest that clathrin-mediated and caveolae-mediated endocytosis of VE-cad contribute to LPS-induced vascular hyperpermeability, although they contribute via different mechanism. The predominant means of endocytosis depends on the time since LPS treatment.

## Introduction

Vascular hyperpermeability, which is induced by endothelial barrier dysfunction, is a common pathogenic process in cases of severe trauma and sepsis and could result in protein-rich tissue edema, abnormalities of the internal environment, abdominal compartment syndrome and multiple organ dysfunction syndrome [1], [2]. Lipopolysaccharide (LPS) is the major pathogenic factor involved in inflammation and sepsis and is an important vascular permeabilizing agent. According to previous reports, LPS regulates endothelial permeability via two main pathways: transcellular and paracellular [3]. The transcellular pathway is the vesicle-mediated transcytosis of macromolecules [4]. The paracellular pathway refers to passage through the intercellular space formed between contacting cells, which leads to the direct leakage of macromolecules [5], and is considered to be the predominant pathway that regulates vascular hyperpermeability compared with the transcellular pathway [6]. At present, the paracellular pathway is thought to be caused by the contraction of stress fibers after LPS stimulation [5]. However, other studies revealed that the polymerization of F-actin and the formation of stress fibers occur only early after LPS treatment; depolymerization occurs after prolonged LPS treatment [7], suggesting that other mechanisms might be involved in this process.

According to the previous studies, interendothelial junctions (IEJs) are important for maintaining the endothelial barrier. Adherens junctions (AJs) are the main type of IEJs between vascular endothelial cells [8], and vascular endothelial cadherin (VE-cad) is an important structural protein of the AJs at the IEJs of vascular endothelial cells [9]. The amount of VE-cad at the plasma membrane could directly modulate the strength of AJs, consequently affecting endothelial barrier function and vascular permeability [10]. Significant downregulation of VE-cad expression at the plasma membrane coincides with LPS-induced hyperpermeability of endothelial cells [11]; however, the specific mechanism through which LPS causes the downregulation of VE-cad and the consequent vascular hyperpermeability remain unknown.

Previous studies have suggested that the expression of cadherin at the plasma membrane and AJs is determined by endocytosis [10] and that the clathrin-mediated endocytosis pathway is generally accepted to be responsible for the internalization of VE-cad and the regulation of hyperpermeability [12], [13]. For example, vascular endothelial growth factor (VEGF)-induced vascular hyperpermeability and the internalization of VE-cad is clathrin-mediated [14]. Some evidence suggests that the internalization of epithelial cadherin (E-cad) could also occur via clathrin-independent, caveolae-mediated pathways in some epithelial tumor cell types, which contributes to the disassembly of AJs and tumor cell invasion [15], [16]. However, it is unknown whether the clathrin-mediated endocytosis of VE-cad and/or the caveolae-mediated endocytosis of VE-cad also contribute to LPS-induced vascular hyperpermeability, whether they contribute in similar or different manners, and which mechanisms might be relevant in these processes.

To address these uncertainties, the internalization of VE-cad through both the clathrin-mediated and caveolae-mediated pathways was observed after LPS treatment of the human vascular endothelial cell line CRL-2922. Specifically, the roles of these pathways in LPS-induced vascular hyperpermeability and the relevant mechanisms were assessed.

## Materials and Methods

### 1. Materials and reagents

The caveolae inhibitor filipin, the antibodies to VE-cad, clathrin, caveolin-1 (Cav-1), and c-Src, clathrin heavy chain siRNA and Cav-1 siRNA were purchased from Santa Cruz Biotechnology (Santa Cruz, CA, USA). The antibody to phospho-caveolin-1 (Tyr14) was obtained from Cell Signaling Technology (Beverly, MA, USA). The antibody to β-actin, the inhibitor of clathrin-mediated endocytosis, chlorpromazine (CPZ), fluorescein isothiocyanate-labeled bovine serum albumin (FITC-BSA), radioimmunoprecipitation assay buffer, FITC-labeled phalloidin, protein G-agarose, and LPS were purchased from Sigma (St. Louis, MO, USA). The cytoskeleton depolymerizing agent cytochalasin D (Cyt D) and the cytoskeleton stabilizer Jasplakinolide (Jasp) were obtained from Enzo Life Sciences (Plymouth Meeting, PA, USA). The FITC-conjugated secondary antibody and rhodamine-conjugated secondary antibody were purchased from Invitrogen (Carlsbad, CA, USA); Dulbecco's modified Eagle's medium (DMEM) was from HyClone Laboratories (Logan, UT, USA). Fetal bovine serum was obtained from Gibco (Grand island, NY, USA), and the 24-well Transwell chambers were from Corning Life Sciences (Lowell, MA, USA). The inhibitor of Src, SU6656, was obtained from Merck KGaA (Darmstadt, Germany). The Toll-like receptor 4 (TLR4) inhibitor CLI-095 was purchased from InvivoGen (San Diego, CA, USA). A plasma membrane protein extraction kit was purchased from Abcam (Cambridge, UK).

### 2. Cell culture

The human vascular endothelial cell line CRL-2922 (ATCC, Manassas, VA, USA) was cultured in DMEM with 4500 mg/L glucose and supplemented with 15% fetal bovine serum in a 5% carbon dioxide cell incubator at 37°C. For the co-immunoprecipitation and western blot analyses, the cells were seeded in 25-mL culture flasks. For the monolayer cell permeability assay, the cells were seeded on polycarbonate membranes in 24-well Transwell chambers. For immunocytochemistry, the cells were seeded on cover slips. The experiments were performed after the cells reached confluence.

### 3. Co-immunoprecipitation

The cells were harvested after treatment with LPS and/or other reagents, and the total protein was extracted. The total protein was incubated with the appropriate primary antibody overnight at 4°C and then with protein G-agarose for 24 h. The immunoprecipitates were washed 4 times using radioimmunoprecipitation assay buffer, eluted by boiling in SDS sample buffer, and separated by routine SDS-polyacrylamide gel electrophoresis (SDS-PAGE). The protein bands of the immunoprecipitation protein and its co-immunoprecipitated objective protein were visualized, and the densitometry data of the protein bands were obtained using Quantity One quantification software (BioRad Laboratories Inc., CA, USA). The degree of co-immunoprecipitation was evaluated by calculating the densitometry ratio of the co-immunoprecipitated objective protein to the corresponding immunoprecipitation protein.

### 4. Western blot and protein expression analysis

For protein expression detection, the cells were harvested after treatment with LPS and/or other reagents, and the total protein was extracted. The total protein was separated by routine SDS-PAGE, and the bands of the objective proteins and β-actin were visualized. The densitometry data of the protein bands were obtained using Quantity One quantification software, and the objective protein expression was evaluated by the densitometry ratio of the objective proteins to β-actin.

For the detection of proteins in the plasma membrane, the cells were harvested after treatment with LPS and/or other reagents, and the protein in the plasma membrane was extracted using the plasma membrane protein extraction kit according to the manufacturer's protocol and a previous report [17]. This extraction involved three cycles of thorough cellular lysis and extensive washing, and only the cytoplasmic protein from the first cycle and the plasma membrane protein from the last cycle were used. The plasma membrane protein was separated by routine SDS-PAGE to visualize the bands of the objective protein, and the cytoplasmic protein was separated by routine SDS-PAGE to visualize the β-actin bands. The densitometry data were obtained using Quantity One quantification software. The objective protein expression at the plasma membrane was evaluated by the densitometry ratio of the objective proteins to β-actin.

### 5. Monolayer cell permeability assay

The Transwell chambers with confluent monolayer endothelial cells were used to examine permeability as previously described [18]. In brief, the cells were treated with LPS and/or other reagents, and FITC-BSA was added to the upper chamber as the tracer. The amount of FITC-BSA that crossed the monolayer to the lower chamber was measured at successive intervals by fluorimetry, and the monolayer cell permeability was reflected by the leakage rate of FITC-BSA.

### 6. Immunocytochemistry and co-localization

After treatment with LPS and/or other reagents, the confluent monolayers were fixed with 4% paraformaldehyde for 30 min at 37°C and were then permeabilized and blocked in a mixture of 0.3% Triton X-100 and 1% BSA for 30 min; the monolayers were then incubated with primary antibodies in supplemented phosphate-buffered saline (PBS) overnight at 4°C. Next, the samples were washed with PBS three times and then incubated with the appropriate FITC- or rhodamine-conjugated secondary antibodies for 1 h at 37°C. Finally, the samples were washed three times with PBS and visualized using a Leica TCS-SP confocal microscope (Wetzlar, Germany). The co-localization of the proteins was assessed by observing the overlapping signals that arose from the different fluorochromes [19], [20].

#### Immunocytochemistry and cytoskeleton structure observation

After treatment with LPS, the confluent monolayers were fixed, permeabilized and blocked according to routine procedures. They were then incubated with FITC-labeled phalloidin for 30 min at 37°C, washed three times with PBS, and visualized on a Leica TCS-SP confocal microscope.

### 7. Statistical analysis

All of the experiments were performed with samples in triplicate or greater, and the data are presented as the mean ± standard deviation (SD) of n observations. The experimental groups were compared by one-way or two-way analysis of variance followed by a post-hoc Tukey's test. Differences were considered statistically significant if the *P* value was <0.05.

## Results

### 1. Clathrin-mediated endocytosis of VE-cad after LPS treatment and its role in vascular hyperpermeability

#### 1.1 VE-cad localization and monolayer cell permeability

**Figure 1:**
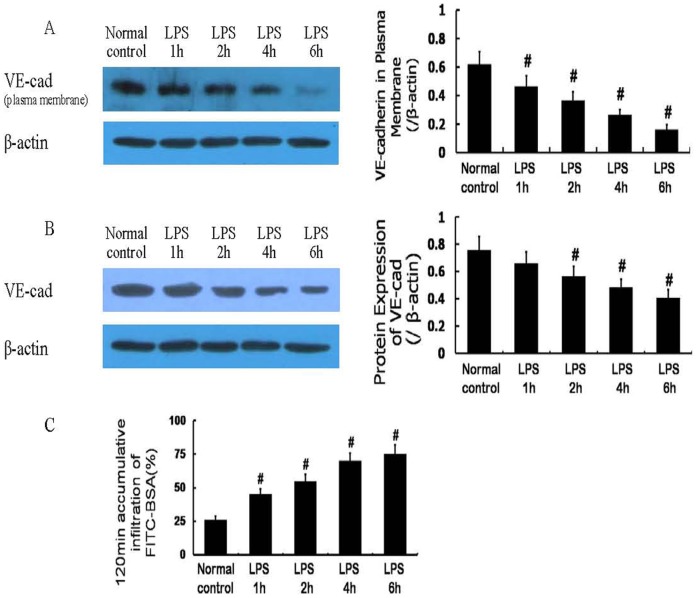
Protein expression of vascular endothelial cadherin (VE-cad) and monolayer cell permeability after lipopolysaccharide (LPS) treatment. The protein expression of VE-cad at the plasma membrane was decreased after LPS treatment (A); the total protein expression of VE-cad was also decreased gradually after LPS treatment (B). The monolayer cell permeability was increased gradually after LPS treatment (C) and was negatively correlated with VE-cad expression, especially VE-cad at the plasma membrane. # *P*<0.05 vs normal control.

Normal cells displayed strong VE-cad expression at the plasma membrane, and decreased plasma membrane VE-cad expression was observed after LPS (10 µg/mL) treatment (Figure 1 A); the total protein expression of VE-cad was also decreased after LPS treatment (Figure 1 B). The permeability of the monolayer cells increased gradually in a time-dependent manner after LPS treatment (Figure 1 C). The monolayer cell permeability and the plasma membrane-localized VE-cad were negatively correlated.

#### Endocytosis of VE-cad via the clathrin-mediated pathway

**Figure 2:**
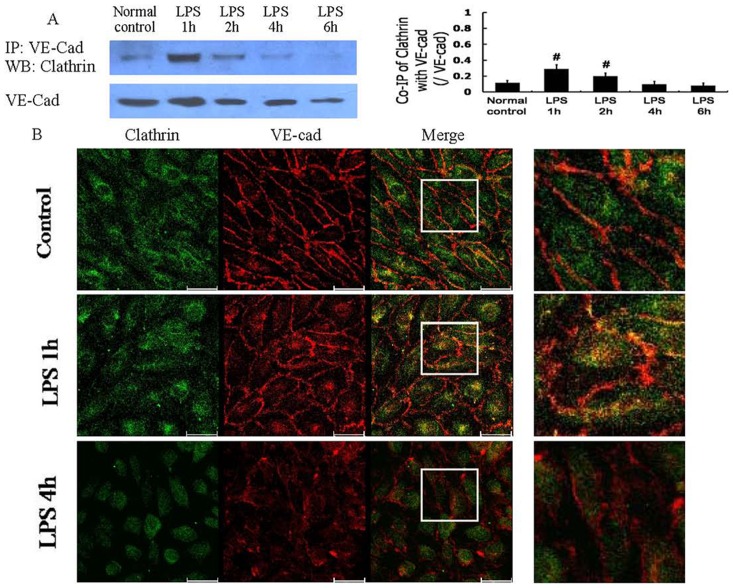
Clathrin-mediated endocytosis of VE-cad after LPS treatment. The co-immunoprecipitation of VE-cad with clathrin was dominant 1 h after LPS treatment (A); immunocytochemistry and confocal microscopy observations also demonstrated the significant co-localization (yellow) of VE-cad with clathrin 1 h after LPS treatment (B). Scale bars: 40 µm. # *P*<0.05 vs normal control group.

The amount of clathrin that co-immunoprecipitated with VE-cad was low in the normal control cells, increased 1 h after LPS treatment, and decreased with time (Figure 2 A). The co-localization of VE-cad and clathrin was altered in a similar pattern (Figure 2 B). The clathrin-mediated endocytosis of VE-cad did not correlate well with the plasma membrane-localized VE-cad or the monolayer cell permeability after LPS treatment.

#### 1.2 Effect of a clathrin-mediated endocytosis inhibitor

**Figure 3:**
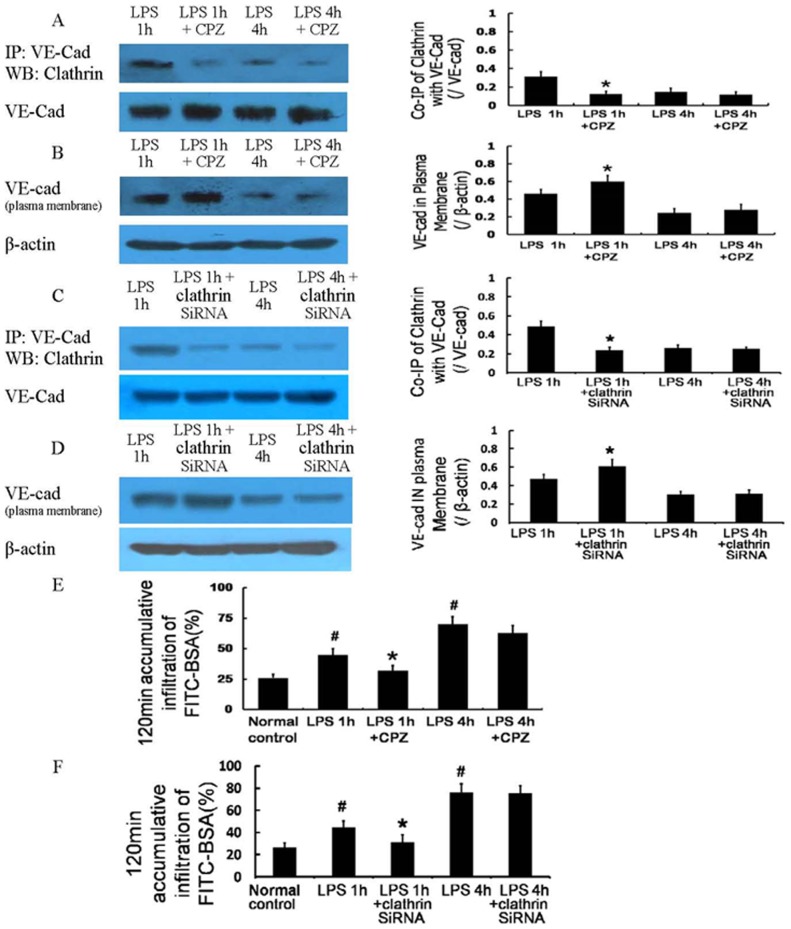
Role of clathrin-mediated endocytosis of VE-cad in LPS-induced vascular hyperpermeability. The clathrin-mediated endocytosis inhibitor chlorpromazine (CPZ) and clathrin siRNA deceased the co-immunoprecipitation of VE-cad with clathrin (A, C), increased the expression of VE-cad at the plasma membrane (B, D), and improved the monolayer cell permeability 1 h after LPS treatment (E, F). # *P*<0.05 vs normal control group. * *P*<0.05 vs 1 h after LPS treatment.

The clathrin-mediated endocytosis inhibitor CPZ (100 µmol/L) decreased the co-immunoprecipitation of VE-cad and clathrin (Figure 3A), increased the localization of VE-cad at the plasma membrane (Figure 3 B), and improved the monolayer cell permeability (Figure 3 E) 1 h after LPS treatment. However, these changes were absent 4 h after LPS treatment. Clathrin siRNA produced similar effects to CPZ in terms of the co-immunoprecipitation of VE-cad and clathrin (Figure 3 C), VE-cad plasma membrane expression (Figure 3 D), and monolayer cell permeability after LPS treatment (Figure 3 F). These results suggest that clathrin-mediated endocytosis of VE-cad occurs 1 h after LPS treatment, leading to a decrease of plasma membrane-localized VE-cad and monolayer cell hyperpermeability. In contrast, the activity of this endocytosis pathway was reduced 4 h after LPS treatment and was not responsible for the internalization of VE-cad and the severe monolayer cell hyperpermeability at that time point.

### 2. Caveolae-mediated endocytosis of VE-cad after LPS treatment and its role in vascular hyperpermeability

#### 2.1 Caveolae-mediated endocytosis of VE-cad

**Figure 4:**
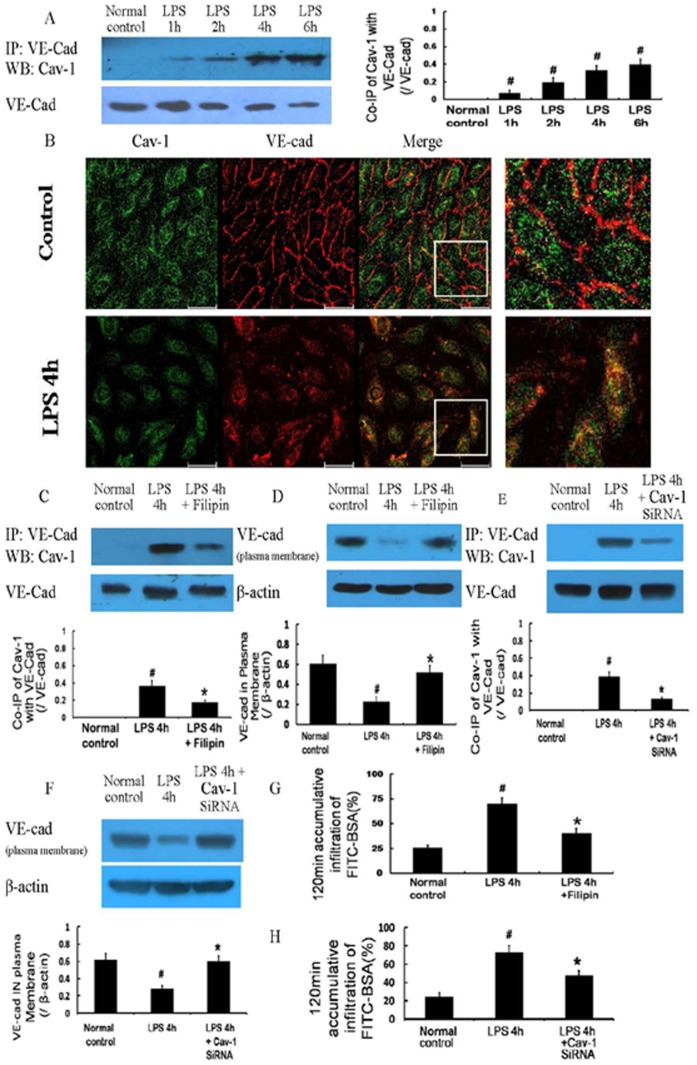
Caveolae-mediated endocytosis of VE-cad after LPS treatment and its role in vascular hyperpermeability. The co-immunoprecipitation of VE-cad with Cav-1, an important structural protein of caveolae, was dominant 4 h after LPS treatment (A); immunocytochemistry and confocal microscopy observations also showed similar results of co-localization (yellow)(B). The caveolae inhibitor filipin and Cav-1 siRNA deceased the co-immunoprecipitation of VE-cad with Cav-1 (C, E), increased the expression of VE-cad at the plasma membrane (D, F), and improved the monolayer cell permeability 4 h after LPS treatment (G, H). Scale bars: 40 µm. # *P*<0.05 vs normal control. * *P*<0.05 vs 4 h after LPS treatment.

The co-immunoprecipitation and co-localization of VE-cad with Cav-1, an important structural protein of caveolae, was negligible in the normal controls and was increased in a time-dependent manner after 10 µg/mL LPS treatment (Figure 4 A, B); it was negatively correlated with the VE-cad at the plasma membrane and positively correlated with monolayer cell permeability after LPS treatment.

#### 2.2 Effect of a caveolae-mediated endocytosis inhibitor

Filipin (5 µg/mL), an inhibitor of caveolae, significantly reduced the co-immunoprecipitation of VE-cad with Cav-1 (Figure 4 C), increased the plasma membrane expression of VE-cad (Figure 4 D), and improved monolayer cell permeability 4 h after LPS treatment (Figure 4 G). The inhibition of Cav-1 by siRNA significantly weakened the caveolae-mediated endocytosis of VE-cad (Figure 4 E), the loss of VE-cad from the plasma membrane (Figure 4 F), and endothelial hyperpermeability (Figure 4 H) 4 h after LPS treatment. These results demonstrate that the caveolae-mediated endocytosis of VE-cad occurs 4 h after LPS treatment and contributes to the internalization of VE-cad, thus aggravating monolayer cell hyperpermeability.

#### 2.3 Activation of signal pathways related to caveolae-mediated endocytosis

**Figure 5:**
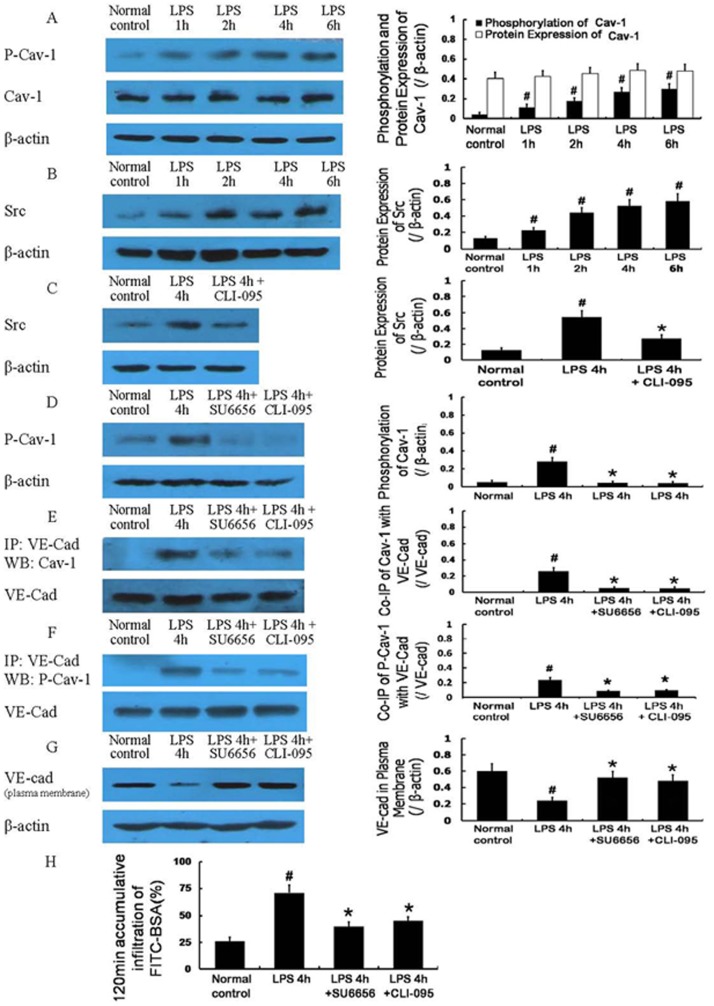
Activation of signaling pathways associated with caveolae-mediated endocytosis after LPS treatment. Protein expression of Cav-1 was not obviously changed after LPS treatment, whereas its phosphorylation (Tyr14) was gradually increased (A). The protein expression of Src was also gradually increased after LPS treatment (B); its increased expression could be decreased by the Toll-like receptor 4 (TLR4) inhibitor CLI-095 (C). The inhibitors of Src and TLR4, SU6656 and CLI-095, respectively, significantly decreased the phosphorylation (Tyr14) of Cav-1 (D), reduced the co-immunoprecipitation of VE-cad with Cav-1 and the co-immunoprecipitation of VE-cad with phospho-Cav-1 (E, F), increased the expression of VE-cad at the plasma membrane (G), and improved the monolayer cell permeability 4 h after LPS treatment (H). P-Cav-1, phospho-Cav-1. # *P*<0.05 vs normal control group. * *P*<0.05 vs 4 h after LPS treatment.

The protein expression of Cav-1 did not significantly change after LPS treatment, whereas the phosphorylation (Tyr14) of Cav-1 showed a significant increase after treatment with LPS (Figure 5 A). LPS also induced a gradual increase in the protein expression of Src (Figure 5 B), and this increased protein expression was decreased by the TLR4 inhibitor CLI-095 (5 µg/mL) (Figure 5 C). The inhibitors of Src and TLR4, SU6656 (2 µmol/L) and CLI-095, respectively, significantly decreased the phosphorylation (Tyr14) of Cav-1 (Figure 5 D), reduced the co-immunoprecipitation of VE-cad with Cav-1 (Figure 5 E) and the co-immunoprecipitation of VE-cad with phospho-Cav-1 (Figure 5 F), increased the plasma membrane expression of VE-cad (Figure 5 G), and improved monolayer cell permeability 4 h after LPS treatment (Figure 5 H).

### 3. Role of the cytoskeleton in the regulation of the two endocytic pathways after LPS treatment

#### 3.1 Cytoskeletal changes

**Figure 6:**
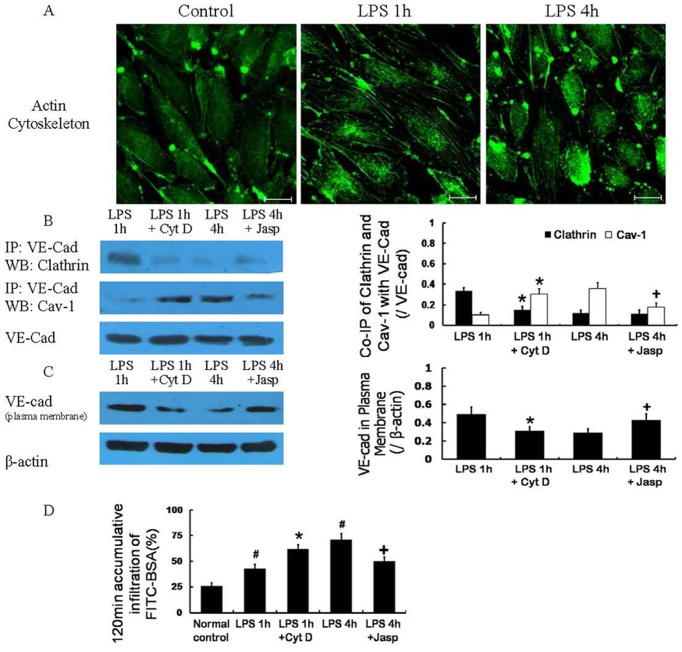
The role of the cytoskeleton in the regulation of two endocytosis pathways after LPS treatment. The cytoskeleton demonstrated a dynamic change after LPS treatment; the F-actin polymerized, and stress fibers formed 1 h after LPS treatment and depolymerized 4 h after LPS treatment (A). The cytoskeleton depolymerizing agent cytochalasin D (Cyt D) could decrease the co-immunoprecipitation of VE-cad with clathrin and increase the co-immunoprecipitation of VE-cad with Cav-1 1 h after LPS treatment (B), and the loss of VE-cad at the plasma membrane (C) and the monolayer cell hyperpermeability (D) 1 h after LPS treatment become more serious. The cytoskeleton stabilizer Jasplakinolide (Jasp) applied 1 h after LPS treatment could decrease the co-immunoprecipitation of VE-cad with Cav-1 4 h after LPS treatment (B), and the location of VE-cad at the plasma membrane (C) and the monolayer cell permeability (D) 4 h after LPS treatment was improved. Scale bars: 20 µm. # *P*<0.05 vs normal control, * *P*<0.05 vs 1 h after LPS treatment. + *P*<0.05 vs 4 h after LPS treatment.

The cytoskeleton showed a dynamic change after LPS treatment. Specifically, 1 h after LPS treatment, F-actin polymerized, and stress fibers formed. However, 4 h after LPS treatment, these fibers depolymerized (Figure 6 A).

#### 3.2 Effect of cytoskeleton regulators

The cytoskeleton-depolymerizing agent Cyt D (2 µmol/L), which was applied 1 h after LPS treatment, could decrease the co-immunoprecipitation of VE-cad with clathrin (Figure 6 B) and increase the co-immunoprecipitation of VE-cad with Cav-1 (Figure 6 B). Therefore, VE-cad was lost from the plasma membrane (Figure 6 C), and monolayer cell hyperpermeability (Figure 6 D) increased.

The cytoskeleton stabilizer Jasp (1 µmol/L), which was applied 1 h after LPS treatment, did not significantly change the co-immunoprecipitation of VE-cad with clathrin (Figure 6 B). In contrast, Jasp decreased the co-immunoprecipitation of VE-cad with Cav-1 4 h after 10 µg/mL LPS treatment (Figure 6 B). Thus, the plasma membrane VE-cad (Figure 6 C) and monolayer cell permeability (Figure 6 D) were improved 4 h after LPS treatment.

## Discussion

The present study focused on the role of the clathrin-mediated and caveolae-mediated endocytosis of VE-cad in LPS-induced vascular hyperpermeability and determined that the clathrin-mediated endocytosis of VE-cad was increased 1 h after LPS treatment and decreased with time. This increase contributed to monolayer cell hyperpermeability early after LPS treatment. In contrast, the caveolae-mediated endocytosis of VE-cad was increased in a time-dependent manner after LPS treatment, which contributed to unremitting and severe monolayer cell hyperpermeability several hours after LPS treatment. The caveolae-mediated endocytosis of VE-cad and the monolayer cell hyperpermeability induced by LPS could be reduced by TLR4 and Src inhibitors. The actin cytoskeleton shifted from polymerization to depolymerization after LPS treatment, and a cytoskeleton-depolymerizing agent could shift VE-cad endocytosis from being clathrin-mediated to being caveolae-mediated. This effect aggravated monolayer cell hyperpermeability 1 h after LPS treatment, whereas the cytoskeleton stabilizer could reduce this shift and reduced monolayer cell hyperpermeability.

These findings indicate that the clathrin-mediated internalization and caveolae-mediated internalization of VE-cad contribute to LPS-induced vascular hyperpermeability, although through different mechanisms. Specifically, the clathrin-mediated endocytosis of VE-cad was dominant early after LPS treatment, which led to the loss of VE-cad plasma membrane localization and monolayer cell hyperpermeability. In contrast, the caveolae-mediated endocytosis of VE-cad was dominant hours after LPS treatment, which induced an unremitting and severe loss of VE-cad plasma membrane localization and monolayer cell hyperpermeability. The caveolae-mediated endocytosis of VE-cad was activated through the LPS-TLR4-Src signaling pathway, and the structural changes in the actin cytoskeleton, specifically from polymerization to depolymerization, were an important cause of the transformation from the clathrin-mediated to caveolae-mediated endocytosis of VE-cad.

Our results provide an explanation for the paradoxical effects observed after LPS treatment. According to previous reports, the paracellular pathway is the predominant pathway that regulates vascular hyperpermeability [6], and the opening of the paracellular pathway mainly results from the contraction of stress fibers after LPS stimulation [5]. Given that F-actin polymerizes and stress fibers form early after LPS treatment and subsequently depolymerize with prolonged LPS stimulation [7], it is unclear how the paracellular pathway is maintained after F-actin polymerization. Our study suggests that although the change in the actin cytoskeleton from polymerization to depolymerization after LPS treatment could weaken the contraction-induced opening of the paracellular pathway, which is mediated by stress fibers, the change could also lead to the caveolae-mediated endocytosis of VE-cad and to the disassembly of AJs. The paracellular pathway is then maintained, regulating vascular hyperpermeability.

The endocytosis of VE-cad is thought to occur mainly via the clathrin-mediated pathway [12], [13]; however, the present study revealed that clathrin-dependent endocytosis of VE-cad was observed 1 h after LPS treatment and gradually decreased with time. This finding is consistent with a previous report demonstrating that the siRNA-mediated knockdown of clathrin could significantly inhibit the internalization of E-cad 10 min after treatment, whereas there was no difference in the internalization of E-cad between the clathrin siRNA-mediated knockdown group and the control group 30 min after treatment in MCF7 cells [21]. From our research, although the timing differed slightly of when clathrin-dependent endocytosis was more predominant, most likely due to the different cell types, the aforementioned report [21] and our study both suggest that cadherin might be endocytosed through a clathrin-independent pathway.

We further observed that VE-cad was endocytosed through the clathrin-independent caveolae-mediated pathway. According to the current literature, E-cad could be endocytosed via the clathrin-independent caveolae-mediated pathway at tumor epithelial cell-cell contacts [16]; however, whether the endocytosis of VE-cad could also occur via the caveolae-mediated pathway remains unknown. Some evidence suggest that caveolae are most likely related to the distribution of VE-cad followed by the disruption of AJs. For example, different concentrations of hydrogen dioxide (0.05–0.8 mmol/L) could induce the phosphorylation (Tyr14) of Cav-1, which is accompanied by the dissociation of AJs in mouse pulmonary vascular endothelial cells [22]; Jens Waschke et al. determined that PMA (an activator of PKC) could disrupt AJs and increase the permeability of wild-type microvascular myocardial endothelial cells, whereas no effect on monolayer integrity was detected in Cav-1(-/-) cells [23]. Our study demonstrates that the internalization of VE-cad could occur via the clathrin-independent caveolae-mediated pathway, which was dominant hours after LPS treatment.

Cav-1, a caveolae scaffolding protein, binds endothelial nitric oxide synthase (eNOS) and negatively regulates eNOS activity and nitric oxide (NO) production to regulate endothelial permeability indirectly [24]; however, the probable role of the Cav-1 phosphorylation-eNOS-NO pathway in LPS-induced endothelial hyperpermeability remains unknown. Previous studies have revealed that eNOS activation is functionally coupled to the phosphorylation of Cav-1 and caveolae internalization; the phosphorylation of Cav-1 (Tyr 14) promoted caveolae-mediated endocytosis and promoted the dissociation of eNOS from caveolin-1 and the simultaneous activation of eNOS [25]–[27]. Therefore, we hypothesized that the caveolae-mediated endocytosis of VE-cad after LPS treatment was accompanied by eNOS activation and NO production. However, previous studies also revealed that the NO produced by eNOS was beneficial for maintaining endothelial barrier function and could exert anti-inflammatory effects within the microcirculation after LPS administration in rats [28], attenuate intestinal microvascular dysfunction and reduce albumin leakage in rats [29], and reverse the increased endothelial permeability induced by IL-1beta, IFN-gamma, and LPS in human brain microvessel endothelial cells [30]. The Cav-1 phosphorylation-eNOS-NO pathway clearly could not be responsible for LPS-induced vascular hyperpermeability and endothelial barrier dysfunction. Hence, the present research did not address caveolae-dependent eNOS activation and NO production; this study focused on another role of caveolae, i.e., endocytosis, and determined that the caveolae-mediated endocytosis of VE-Cad contributed to LPS-induced endothelial hyperpermeability.

Furthermore, our discovery of the crosstalk between the paracellular and transcellular pathways during the regulation of LPS-induced vascular hyperpermeability is also noteworthy. The paracellular and transcellular pathways are routinely considered the primary, independent mechanisms of LPS-induced hyperpermeability of continuous endothelial cells. The paracellular pathway allows fluid and micromolecular solutes to pass freely from the blood plasma into the interstitial space and also allows the passage of albumin and other macromolecules in some pathological conditions. In contrast, the transcellular pathway allows macromolecules (e.g., albumin) to be transported in a vesicle-dependent manner from the lumen of blood vessels to tissue spaces [31]. Our study determined that caveolae, an important vesicle in the transcellular pathway, could internalize VE-cad, an important gating macromolecule in the paracellular pathway, which resulted in the opening of the paracellular pathway and vascular hyperpermeability. These findings suggest that although the paracellular pathway is considered the predominant pathway for vascular hyperpermeability, the transcellular pathway might play an important role in the opening of the paracellular pathway in certain conditions.

Based on the data from previous studies, we hypothesized that changes in the actin cytoskeleton could regulate if VE-cad endocytosis was clathrin-mediated or caveolae-mediated after LPS treatment. Asa Engqvist-Goldstein et al. reported that the disruption of the cortical actin cytoskeleton by genetic approaches or chemical methods inhibited clathrin-mediated endocytosis in yeast [32]. Similarly, Defne Yarar et al. determined that an actin cytoskeleton-depolymerizing agent, latrunculin A, resulted in an 80% reduction in the formation of clathrin-coated vesicles in mammalian cells [33]. However, the depolymerization of the actin cytoskeleton with latrunculin A triggers the rapid and massive movement of caveolin-positive structures toward the centrosomal region of the cell [34]. Le Shen et al. observed that latrunculin A-induced actin depolymerization disrupts the structure and function of tight junctions (TJs) in Madin-Darby canine kidney cells via the caveolae-mediated endocytosis of TJ components [35]. In this study, we demonstrated that the actin cytoskeleton polymerized and stress fibers formed at the early stages after LPS administration. Accordingly, the clathrin-mediated pathway was responsible for most of the endocytosis of VE-cad. Subsequently, the actin cytoskeleton depolymerized significantly, and the clathrin-mediated endocytosis of VE-cad reduced gradually. In contrast, the caveolae-mediated internalization of VE-cad began and increased in a time-dependent manner. The detailed mechanism regarding how the actin cytoskeleton mediates these effects remains unclear, although it might be related to the structural and mechanical function of the cytoskeleton, which could affect the localization of the endocytic machinery, the invagination and separation of nascent endocytic vesicles, and the translocation of endocytic vesicles from the plasma membrane into the cytoplasm [32], [36]–[38]. Further studies are required to fully understand this process.

In the present study, the signaling pathway that activated caveolae-mediated endocytosis after LPS treatment was assessed. We observed a significant increase in the phosphorylation (Tyr14) of Cav-1 after LPS treatment, and this event was positively correlated with the caveolae-mediated endocytosis of VE-cad. Furthermore, in human lung microvascular endothelial cells, the activation of Src could induce the caveolae-mediated endocytosis of albumin and cholera toxin subunit B through Cav-1 phosphorylation (Tyr14) [32]. Because LPS also leads to the activation of Src via the classical signaling pathway mediated by TLR4 [39]–[40], we hypothesized that LPS could induce caveolae-mediated endocytosis through TLR4 and Src. The data reported in this study confirmed that the caveolae-mediated endocytosis of VE-cad and monolayer cell hyperpermeability were activated through the LPS-TLR4-Src signaling pathway. However, further research is needed to understand the relationship between this signaling pathway and cytoskeleton depolymerization during the initiation of LPS-induced caveolae-mediated endocytosis.

The present study also demonstrated that the loss of VE-cad plasma membrane localization and monolayer cell hyperpermeability differed in response to clathrin-mediated and caveolae-mediated internalization; however, the reason for this difference is unclear. According to previous studies, Rab11, a marker of the recycling endosome, is also a marker of the clathrin-mediated endocytosis pathway [41]. E-cad travels to Rab11-positive recycling endosomes after being internalized and might return to the cell surface to construct new AJs [36]; the VE-cad that was internalized via the clathrin-mediated endocytosis pathway after VEGF treatment could relocalize to the cell surface and be incorporated into cell-cell contacts after VEGF removal [14]. In contrast, polyglycol microparticles that are internalized via caveolae-mediated endocytosis co-localize with LAMP2, which is a marker of late endosomes/lysosomes [42]; an inhibitor of caveolae-mediated endocytosis could significantly reduce the entry of fibronectin into lysosomes and reduce its degradation in myofibroblasts [43]. These reports facilitate the understanding that the internalization of VE-cad via clathrin-mediated endocytosis might be relevant to the entry of VE-cad into Rab11-positive recycling endosomes, and this feature might be reversible. Therefore, the loss of VE-cad plasma membrane localization and vascular hyperpermeability were limited. Although the caveolae-mediated endocytosis of VE-cad might be relevant to its entry into lysosomes and relevant to degradation, which explains why the loss of VE-cad plasma membrane localization and vascular hyperpermeability were severe. However, further research is necessary to verify this hypothesis.

The present study suggests that the clathrin-mediated and caveolae-mediated endocytosis pathways participate in the plasma membrane loss of VE-cad and vascular hyperpermeability after LPS treatment, although these endocytosis pathways participate via different mechanism. Specifically, clathrin-mediated endocytosis is dominant early after LPS treatment, and caveolae-mediated endocytosis is dominant hours after LPS treatment. The caveolae-mediated endocytosis of VE-cad is activated through the LPS-TLR4-Src signal pathway. Structural changes in the actin cytoskeleton regulate the switch between the clathrin-mediated and caveolae-mediated endocytosis of VE-cad.
